# Survival factor 1 contributes to the oxidative stress response and is required for full virulence of *Sclerotinia sclerotiorum*

**DOI:** 10.1111/mpp.12801

**Published:** 2019-05-09

**Authors:** Yang Yu, Jiao Du, Yabo Wang, Mengyao Zhang, Zhiqiang Huang, Junsong Cai, Anfei Fang, Yuheng Yang, Ling Qing, Chaowei Bi, Jiasen Cheng

**Affiliations:** ^1^ College of Plant Protection Southwest University Chongqing City 400715 P R China; ^2^ State Key Laboratory of Agricultural Microbiology Huazhong Agricultural University Wuhan City 430070 P R China

**Keywords:** oxidative stress, *Sclerotinia sclerotiorum*, Survival factor 1, virulence

## Abstract

*Sclerotinia sclerotiorum* is a devastating necrotrophic fungal pathogen that infects over 400 species of plants worldwide. Reactive oxygen species (ROS) modulations are critical for the pathogenic development of *S. sclerotiorum*. The fungus applies enzymatic and non‐enzymatic antioxidants to cope with the oxidative stress during the infection processes. Survival factor 1 was identified and characterized to promote survival under conditions of oxidative stress in *Saccharomyes cerevisiae*. In this research, a gene named *SsSvf1* was predicted to encode a survival factor 1 homologue in *S. sclerotiorum*. *SsSvf1* transcripts showed high expression levels in hyphae under oxidative stress. Silencing of *SsSvf1* resulted in increased sensitivity to oxidative stress in culture and increased levels of intracellular ROS. Transcripts of *SsSvf1* showed a dramatic increase during the initial stage of infection and the gene‐silenced strains displayed reduced virulence on oilseed rape and *Arabidopsis thaliana*. Inhibition of plant ROS production partially restores virulence of *SsSvf1* gene‐silenced strains. *SsSvf1* gene‐silenced strains exhibited normal oxalate production, but were impaired in compound appressorium formation and cell wall integrity. The results suggest that *SsSvf1* is involved in coping with ROS during fungal‐host interactions and plays a crucial role in the pathogenicity of *S. sclerotiorum*.

## Introduction

*Sclerotinia sclerotiorum* (Lib.) de Bary is one of the most devastating fungal pathogens with a worldwide distribution. This pathogen can infect more than 400 plant species and lead to significant losses in many cultivated crops including oilseed, sunflower, soybean and the common bean (Boland and Hall, 1994; Bolton *et al*., 2006; Purdy, 1979).

*S. sclerotiorum* has been considered a model necrotrophic fungal pathogen, which kills host tissue via the secretion of oxalic acid (OA) (Cessna *et al*., 2000; Favaron *et al*., 2004; Kim *et al*., 2008; Williams *et al*., 2011) and cell wall degrading enzymes (Martel *et al*., 1996; Poussereau *et al*., 2001; Riou *et al*., 1991; Yajima *et al*., 2009; Yu *et al*., 2016; Zuppini *et al*., 2005). Recent evidence indicates that this fungus secretes effector proteins to suppress host defence in the initial stage of infection (Guyon *et al*., 2014; Lyu *et al*., 2016; Yang *et al*., 2018; Zhu *et al*., 2013). These strategies are mainly applied by biotrophic and hemibiotrophic fungal pathogens. In addition, biotrophic growth at the leading edge of fungal colonization was suggested for *S. sclerotiorum* (Kabbage *et al*., 2015). These studies indicate that the pathogenesis of *S. sclerotiorum* is more complex than we thought and more evidence is needed to detail the underlying molecular mechanism.

Rapid generation of reactive oxygen species (ROS) including hydrogen peroxide (H_2_O_2_), the superoxide anion (O_2_^−^), and hydroxyl radical (OH•) is an early resistance response in many plant/pathogen interactions (Lamb and Dixon, 1997). Such oxidative bursts have direct and powerful antimicrobial activity including inhibition of the spore germination of a number of fungal pathogens (Mousavi and Robson, 2004; Peng and Kuc, 1992). In response, fungal pathogens apply specific enzymes and non‐enzyme‐mediated antioxidant mechanisms to handle ROS (Aguirre *et al*., 2006). Several studies have shown that ROS modulation is critical for the hyphae of *S. sclerotiorum* to successfully colonize host plant tissue (Kim *et al*., 2011; Williams *et al*., 2011; Yarden *et al*., 2014); however, evidence for the molecular mechanisms of ROS detoxification and tolerance in *S. sclerotiorum* are still sparse.

The Survival factor 1 (*SVF1*) gene was first identified in *Saccharomyces cerevisiae* in a screen for mutations that could be functionally complemented by exogenous expression of the human anti‐apoptotic gene *Bcl‐x_L_* (Brace *et al*., 2005; Vander Heiden *et al*., 2002). However, *SVF1* and *Bcl‐x_L_* have distinct roles in regulating cell survival (Brace *et al*., 2005). *S. cerevisiae* cells lacking Svf1 protein showed hypersensitivity to direct chemical precursors of ROS, suggesting that *SVF1* is necessary for survival under oxidative stress (Brace *et al*., 2005). Deeper research has shown that Svf1‐mediated cell survival under conditions of oxidative stress by affecting the sphingolipid metabolism in *S. cerevisiae* (Brace *et al*., 2007). To date, the role of Svf1 in the oxidative stress response and pathogenicity of filamentous fungal pathogens has remained unknown.

Here, a gene in *S. sclerotiorum* (SS1G_01919) named *SsSvf1* (*Sclerotinia sclerotiorum* Survival factor 1) was predicted to encode a yeast Svf1 homologous protein. The function of *SsSvf1* was determined via a reverse genetic approach, and its role in oxidative stress response and pathogenicity was investigated. The research may help clarify the function of Svf1 in fungal plant pathogens and the pathogenicity of *S. sclerotiorum* in more detail.

## Results

### *SsSvf1* encodes a survival factor‐1 homologue in *S. sclerotiorum*

**Figure 1:**
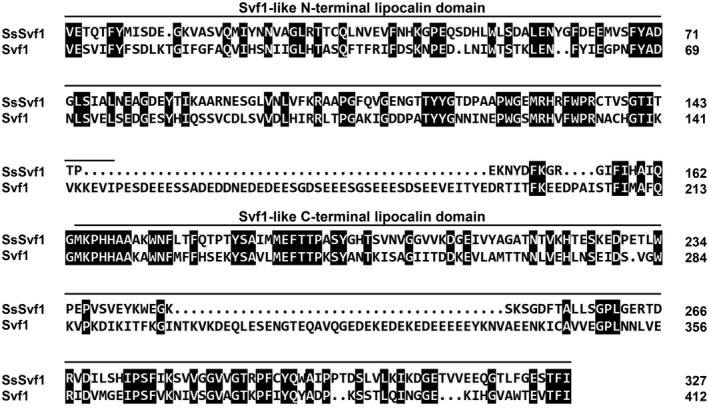
Alignment of amino acid sequences of Svf1 domains in SsSvf1 and yeast Svf1 protein (KZV12585) using ClustalX. Shading indicates sequence similarities of 100% (dark).

The *S. sclerotiorum SsSvf1* gene consists of four exons and three introns, and encodes a protein with 381 amino acids. Conserved Domain Database (CDD) analysis of the protein sequence revealed that a Svf‐like domain was predicted at amino acid position T^52^–I^380^ (Marchler‐Bauer *et al*., 2017). Alignment of amino acid sequences of the N‐terminal and C‐terminal of the Svf1 domains in SsSvf1 and yeast Svf1 exhibit great similarity (Brace *et al*., 2005) (Fig. 1). BLASTP searches using the amino acid sequence of SsSvf1 as a query showed that the homologous sequences are widely present in fungi including some important plant pathogens such as *Botrytis cinerea* (XP_001548941), *Gibberella zeae* (XP_011323561) and *Colletotrichum higginsianum* (XP_018161001).

### *SsSvf1* is required for response to oxidative stress

**Figure 2:**
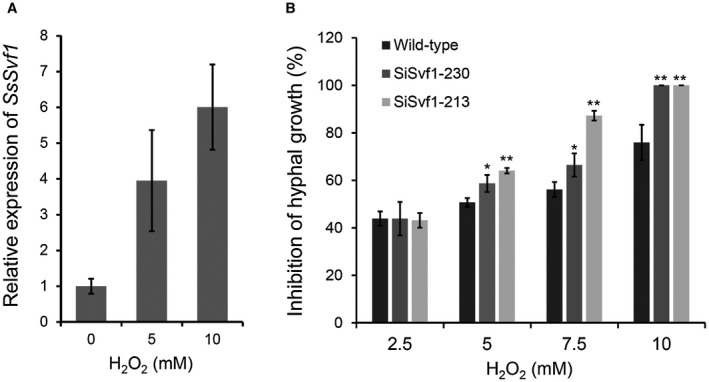
Functional analysis of *SsSvf1* in response to H_2_O_2_. (A) Relative expression level of *SsSvf1* in hyphae treated with H_2_O_2 _(5 mM and 10 mM). Total *SsSvf1* cDNA abundance in the samples was normalized using *tub1* gene as a control. The relative expression of *SsSvf1* in the untreated strain was set as one. Bars indicate standard deviation. (B) Percent growth inhibition of wild‐type strain and *SsSvf1* gene‐silenced strains on potato dextrose agar (PDA) medium with H_2_O_2_. The strains were inoculated on PDA plates amended with H_2_O_2 _at concentrations of 0 mM to 10 mM. Percentage inhibition of hyphal growth was calculated at 36 hpi. Bars indicate standard deviation. Asterisks denote significant differences (one‐way analysis of variance [ANOVA]): *P < 0.05; **P < 0.01.

The expression of *SsSvf1* under oxidative stress conditions was analysed to explore the role of the *SsSvf1* gene in response to oxidative stress of *S. sclerotiorum*. As shown in Fig. 2A, the expression level of *SsSvf1* was much higher in hyphae treated with H_2_O_2 _(5 mM and 10 mM). To determine the function of *SsSvf1*, a gene‐silencing vector was constructed based on pSilent‐1 (Nakayashiki *et al*., 2005) as described in methods. The vector was linearized and transformed into the wild‐type strain of *S. sclerotiorum* via PEG (polyethylene glycol) methods (Rollins, 2003). Several transformants were obtained, and silencing of *SsSvf1* in the transformants was evaluated by real‐time Reverse Transcription‐Polymerase Chain Reaction (RT‐PCR) (Fig. S1). The expression levels of *SsSvf1* in SiSvf1‐230 and SiSvf1‐213 were 15% and 2% of that in the wild‐type strain, respectively. Thus, these two strains were chosen for deeper research.

**Figure 3:**
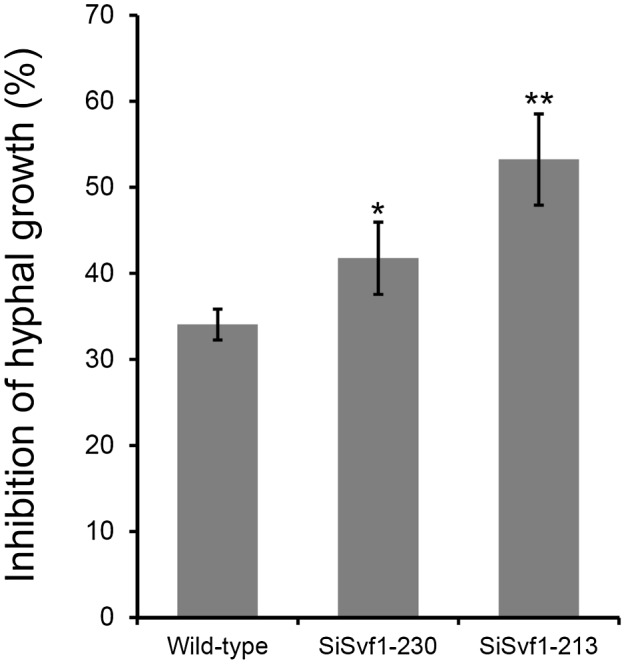
The inhibition of menadione to the hyphal growth of *SsSvf1* gene‐silenced strains and wild‐type strain. Bars indicate standard deviation. Asterisks denote significant differences (one‐way analysis of variance [ANOVA]): *P < 0.05; **P < 0.01.

The hyphal growth of the wild‐type and *SsSvf1* gene‐silenced strains on potato dextrose agar (PDA) containing 0 mM to 10 mM H_2_O_2_ were compared. The results showed that these two gene‐silenced strains displayed wild‐type levels of susceptibility to 2.5 mM H_2_O_2_, while being more sensitive at higher H_2_O_2_ concentrations (Fig. 2B). SiSvf1‐230 and SiSvf1‐213 were also more sensitive to menadione, a chemical inducer of oxidative stress, than the wild‐type strain (Fig. 3). The results indicated that *SsSvf1* was required for managing oxidative stress in *S. sclerotiorum*.

### *SsSvf1* gene‐silenced strains show overproduction of ROS

**Figure 4:**
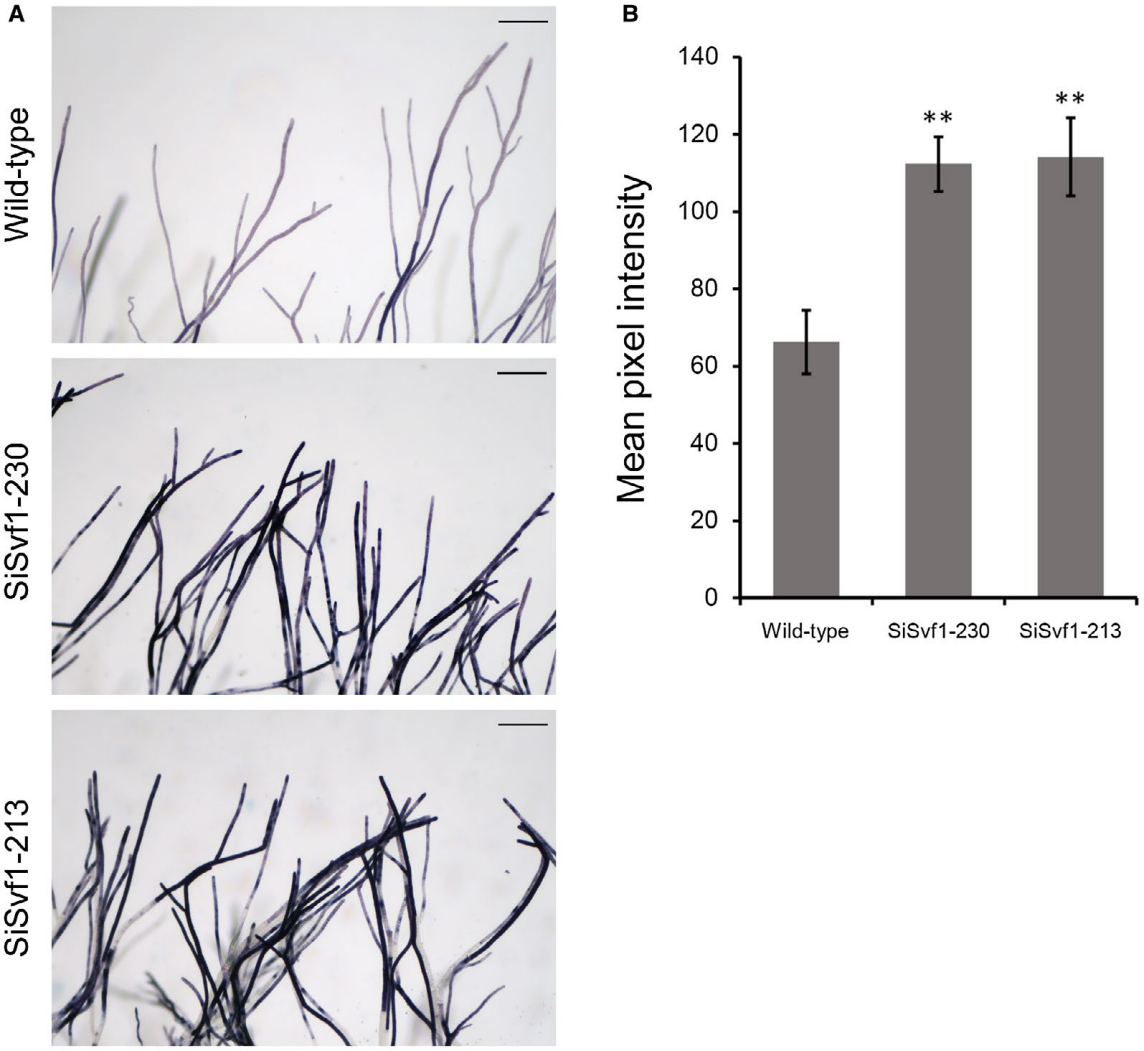
Reactive oxygen species (ROS) accumulation in *SsSvf1* gene‐silenced strains. (A) Detection of ROS production in the hyphae of wild‐type and *SsSvf1* gene‐silenced strains. The strains were inoculated on potato dextrose agar (PDA) plates and stained with 0.5 mg/mL nitroblue tetrazolium (NBT) at 2 dpi. Photographs were taken with light microscopy. Blue staining indicates the accumulation of superoxide. Bars = 200 μM. (B) Mean pixel intensity in the hyphal tips stained with NBT to detect superoxide. Bars indicate standard deviation. Asterisks denote significant differences (one‐way analysis of variance [ANOVA]): **P < 0.01.

Svf1 inhibits ROS generation in *S. cerevisiae* (Brace *et al*., 2005). To understand whether silencing of *SsSvf1* would lead to altered ROS generation, we detected ROS production during hyphal growth in *SsSvf1* gene‐silenced strains. The strains were stained with nitroblue tetrazolium (NBT), which specifically detects superoxide. More dark‐blue formazan precipitates were seen in the hyphae of SiSvf1‐230 and SiSvf1‐213, which indicated that *SsSvf1* gene‐silenced strains produce more superoxide than the wild‐type strain (Fig. 4A). The ROS production in the hyphae tips was then quantified via mean pixel intensity. The results showed that SiSvf1‐230 and SiSvf1‐213 exhibited greatly increased superoxide production (Fig. 4B).

### *SsSvf1* gene‐silenced strains show reduced virulence and a lower efficiency of appressoria formation

**Figure 5:**
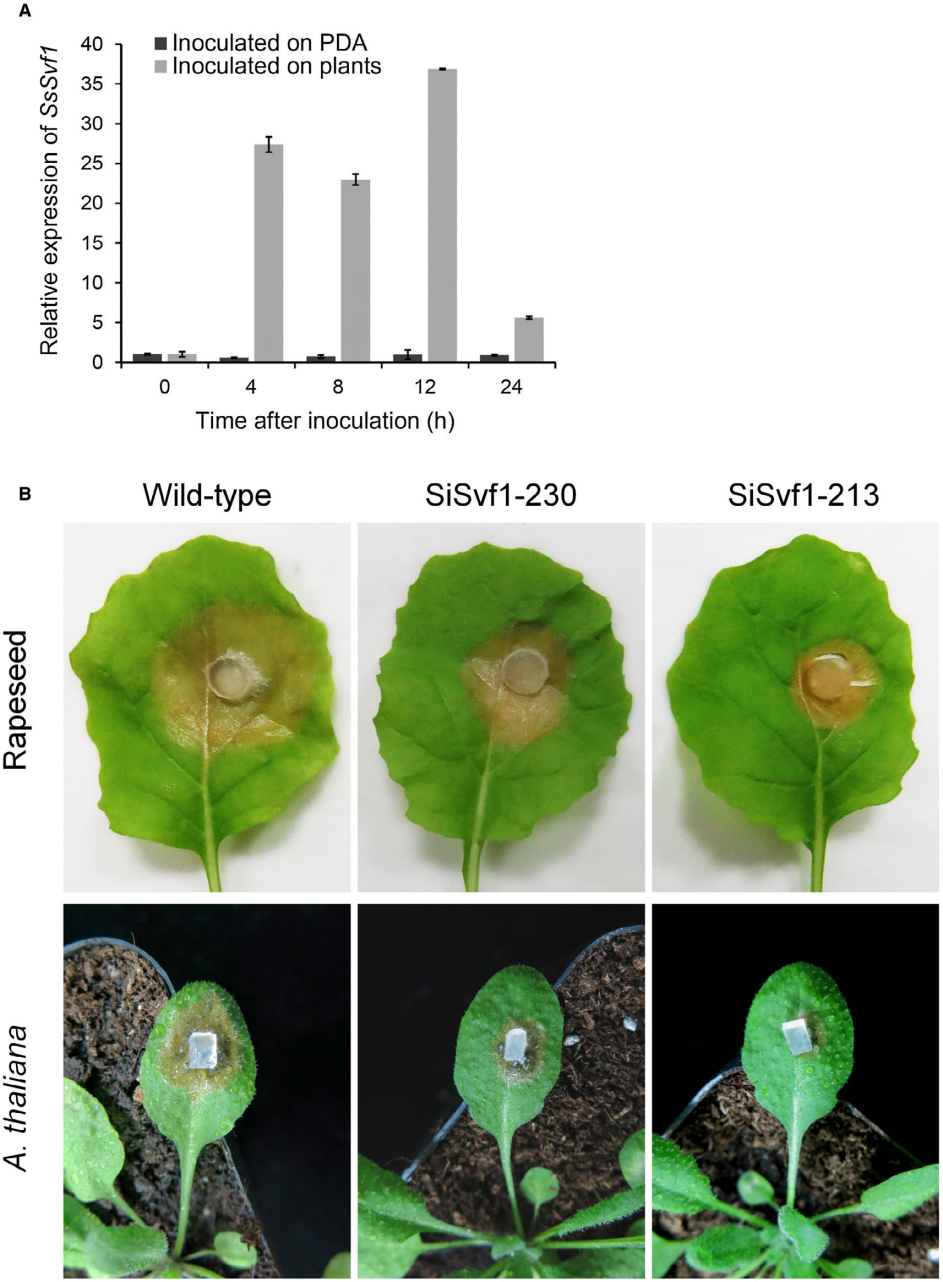
Functional characterization of *SsSvf1* in pathogenicity of *S. sclerotiorum*. (A) Relative expression of *SsSvf1* in wild‐type hyphae after contact with *Arabidopsis thaliana* and growing on potato dextrose agar (PDA) plates. The *Tub1* gene in each sample was used as an internal control. The relative expression of *SsSvf1* in hyphae stage or in hyphae inoculated on plants at 0 h was set as one. Bars indicate standard deviation. (B) Pathogenicity of *SsSvf1* gene‐silenced strains on detached leaves of rapeseed and on *A. thaliana* plants. Each strain was investigated with five rapeseed leaves or *A. thaliana* plants each time. One representative replicate from three experiments is shown.

The expression level of *SsSvf1* during wild‐type infection was evaluated, and the results indicated that the expression of *SsSvf1* showed a strong increase during the initial stage of infection (Fig. 5A). To carry out functional analysis of the role of *SsSvf1* in the pathogenicity of *S. sclerotiorum*, *SsSvf1* gene‐silenced strains were inoculated on detached oilseed rape leaves and on *Arabidopsis thaliana* plants. As shown in Fig. 5B, both gene‐silenced strains caused small lesions on the oilseed rape leaves. The smaller lesions were also observed on *A. thaliana* inoculated with the *SsSvf1* gene‐silenced strains (Fig. 5B). The results indicate that *SsSvf1* is important for the pathogenicity of *S. sclerotiorum*.

**Figure 6:**
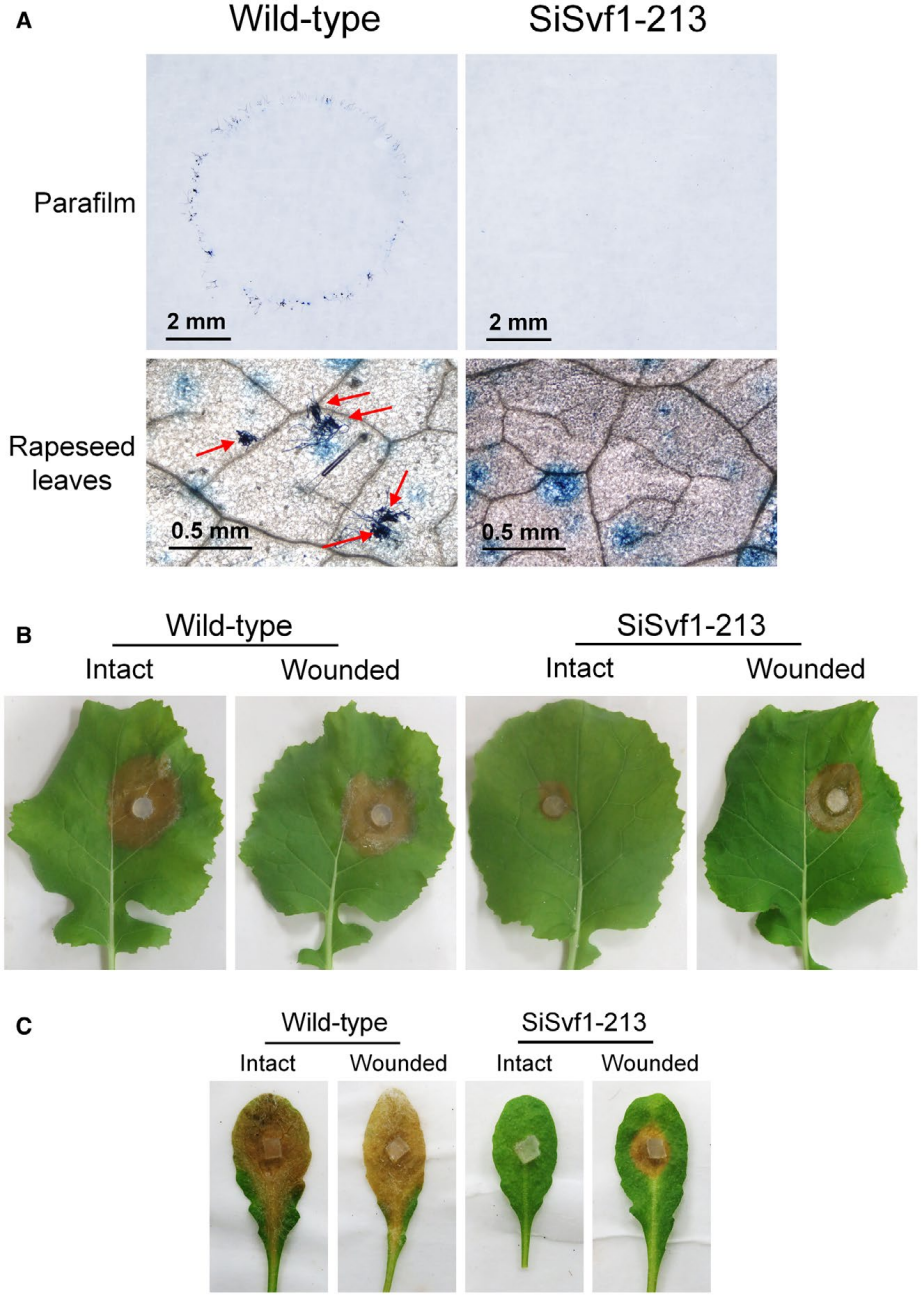
Appressoria formation of *SsSvf1* gene‐silenced strains. (A) Compound appressoria formation of wild‐type strain and SiSvf1‐213 on parafilm and on rapeseed leaves inoculated with mycelial plugs. The photographs were taken at 8 hpi. Arrowheads indicate appressoria formation. (B) Pathogenicity of wild‐type strain and SiSvf1‐213 on wounded leaves of rapeseed. (C) Pathogenicity assay on wounded leaves of *A. thaliana*. Photographs were taken at 48 hpi. One representative replicate from the three experiments is shown.

The OA and compound appressoria were key factors in the pathogenesis of *S. sclerotiorum* infection; thus, the OA accumulation and compound appressorium formation were compared between the wild‐type and *SsSvf1* gene‐silenced strains. Our results showed that *SsSvf1* gene‐silenced strains secreted similar levels of OA as the wild‐type strain (Fig. S2). To assay appressoria formation, both the wild‐type and *SsSvf1* gene‐silenced strains were inoculated on parafilm‐overlaid growth medium and on rapeseed leaves. The results showed that the wild‐type strain formed complex and frequent appressoria, but SiSvf1‐213 rarely produced appressoria on parafilm or rapeseed leaves (Fig. 6A). This indicated that *SsSvf1* is associated with compound appressoria formation in *S. sclerotiorum*. To confirm this, the rapeseed leaves were wounded with a dissecting needle and then inoculated with the wild‐type strain and SiSvf1‐213. The results showed that SiSvf1‐213 caused larger lesions by 48 h post‐inoculation on wounded rapeseed leaves than on intact leaves (Fig. 6B). SiSvf1‐213 was also inoculated on wounded leaves of *A. thaliana* and similar phenotypes were observed (Fig. 6C).

### Inhibition of plant ROS production partially restores virulence of *SsSvf1* gene‐silenced strain

**Figure 7:**
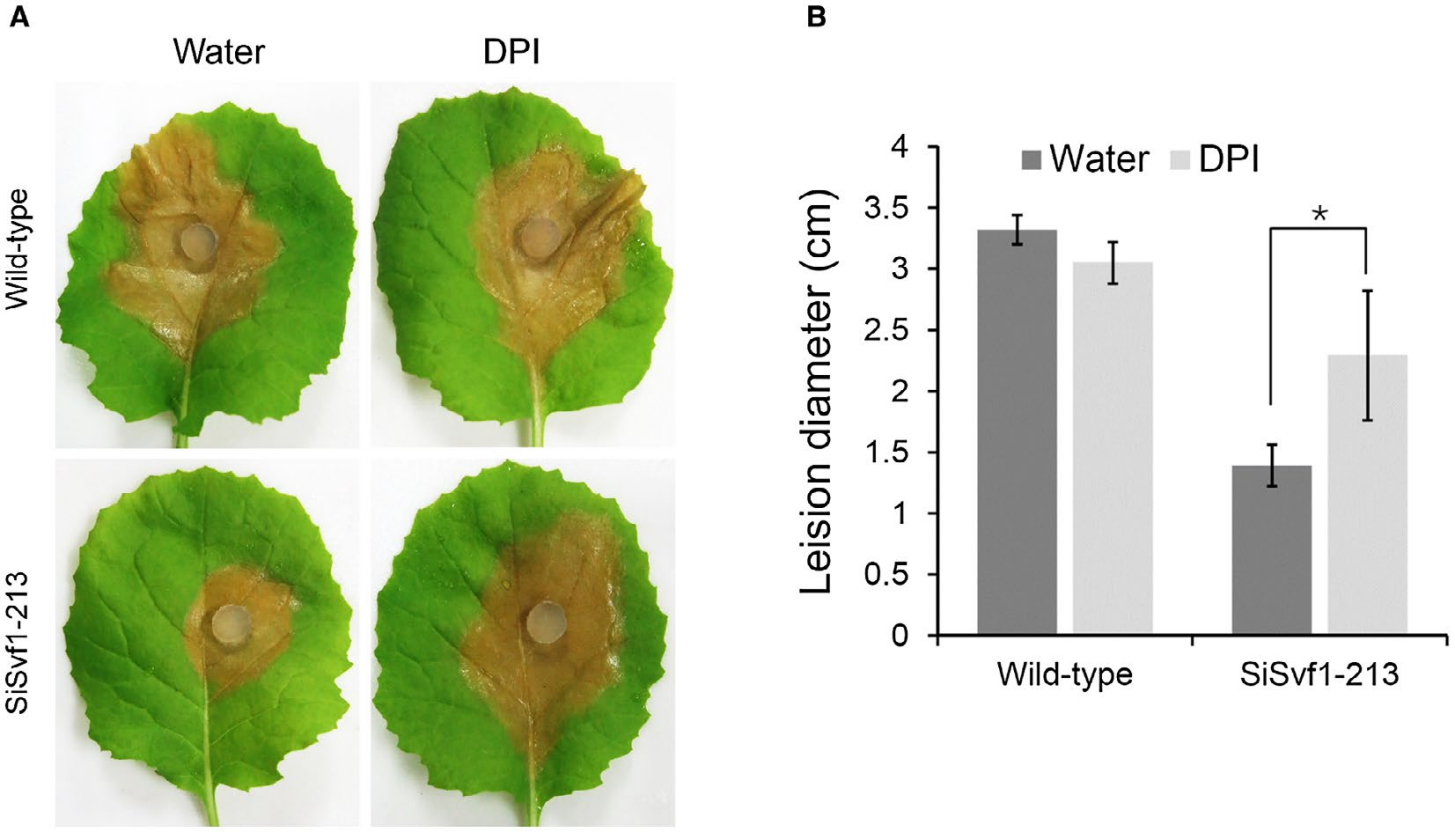
Pathogenicity of *SsSvf1* gene‐silenced strains restored by diphenyleneiodonium (DPI) treatment. (A) Pathogenicity assay. The rapeseed leaves were inoculated with mycelium plugs of wild‐type strain and SiSvf1‐213 after treatment with or without DPI (5 μM) over three independent experiments. Photographs were taken at 48  hpi and the figure shows representative photographs. (B) The size of the expending lesions. Bars indicate standard deviation. Asterisks denote significant differences (one‐way analysis of variance [ANOVA]): *P < 0.05.

*SsSvf1* gene‐silenced strains showed increased sensitivity to ROS, which is an early plant response to pathogen infection. Thus, the virulence of *SsSvf1* gene‐silenced strain was next tested on oilseed rape leaves with reduced generation of ROS. The rapeseed leaves were sprayed with diphenyleneiodonium (DPI) to inhibit activity of the plant NADPH oxidases, and the leaves were then inoculated with the wild‐type strain and SsSvf1‐213, respectively. As shown in Fig. 7, larger lesions were produced by SiSvf1‐213 in leaves treated with DPI than in leaves treated with water.

### *SsSvf1* gene‐silenced strains are impaired in cell wall integrity of hyphae

**Figure 8:**
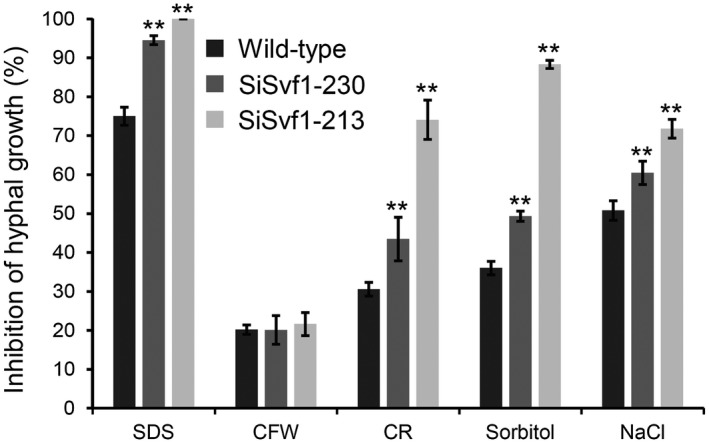
Sensitivity of *SsSvf1* gene‐silenced strains to cell wall perturbation agents and hyperosmotic stress. The strains were inoculated on potato dextrose agar (PDA) plates amending 0.02% sodium dodecyl sulphate (SDS), 200 μM calcofluor white (CFW), 0.4 g/L Congo red (CR), 1 M sorbitol, and 0.4 M NaCl. Percentage inhibition of hyphal growth was calculated at 36 hpi. Bars indicate standard deviation. Asterisks denote significant differences (one‐way analysis of variance [ANOVA]): **P < 0.01.

The cell wall integrity was compared between the wild‐type and *SsSvf1* gene‐silenced strains. The results showed that SiSvf1‐230 and SiSvf1‐213 were more sensitive to SDS (sodium dodecyl sulphate) than the wild‐type strain, suggesting a weakened cell wall for *SsSvf1* gene‐silenced strains (Fig. 8). To evaluate cell wall alteration, the effects of specific cell wall perturbation agents to the *SsSvf1* gene‐silenced strains were determined. The results showed that SiSvf1‐230 and SiSvf1‐213 displayed wild‐type levels of susceptibility to calcofluor white (CFW) while being more sensitive to Congo red (CR) (Fig. 8). This suggests that *SsSvf1* gene‐silenced strains were impaired in some aspect of hyphal cell wall integrity. Since the cell wall integrity pathway and hyperosmotic stress response share common functional aspects and are positively coordinated (Alonso‐Monge *et al*., 2001; Rodríguez‐Peña *et al*, 2010), the hyphal growth of *SsSvf1* gene‐silenced strains under hyperosmotic stress were determined. The inhibition of hyphal growth was significantly greater for SiSvf1‐230 and SiSvf1‐213 than the wild‐type strain when growing on medium with sodium chloride or sorbitol (Fig. 8).

## Discussion

Fungi use specific enzymatic and non‐enzymatic processes to regulate antioxidant response (Aguirre *et al*., 2006). To date, only a few proteins have been reported related to the anti‐oxidative stress responses in *S. sclerotiorum*. *SsSOD1* encodes a Cu/Zn superoxide dismutase (SOD) in *S. sclerotiorum*, and the gene‐deletion mutant exhibited increased sensitivity to oxidative stress (Xu and Chen, 2013). Our previous report also showed that a putative BAX inhibitor‐1 in *S. sclerotiorum* was involved in withstanding H_2_O_2_ (Yu *et al*., 2015). In this study, *SsSvf1* is predicted to encode a *S. cerevisiae* Svf1 homologue in *S. sclerotiorum. SsSvf1* shows increased expression under oxidative stress and the gene‐silenced strains are more sensitive to H_2_O_2 _and menadione. To date, Svf1 was only identified and characterized in *S. cerevisiae*; it was required for survival in response to oxidative stress (Brace *et al*., 2005). Our data demonstrates the function of SVF1 homologues in filamentous fungi for the first time and suggests an important role of Svf1 in response to oxidative stress in *S. sclerotiorum*.

Evidence for the molecular mechanism underlying Svf1 function in oxidative stress response is still sparse. *S. cerevisiae SVF1* gene expression in mammalian cells increases apoptotic resistance (Brace *et al*., 2005). Deep research showed that yeast Svf1 regulates cell survival by affecting sphingolipid metabolism (Brace *et al*., 2007). Sphingolipids are important regulators of apoptosis in plant and animal cells and some sphingolipid metabolites may promote cell survival following induction of death by various treatments (Alden *et al*., 2011; Maceyka *et al*., 2002). In this research, the relationship between *SsSvf1* and sphingosine metabolism in *S. sclerotiorum* is unclear. The sequences of SsSvf1 were used in a search for potential structural homologues via the HHpred server (Söding *et al*., 2005; Zimmermann *et al*., 2018). The top‐ranked hit is a putative lipocalin in *Nitrosomonas europaea* with an E‐value of 4.94e‐24 and a probability score of 99.91. A search with WoLF PSORT server showed that SsSvf1 is most likely located in the cytoplasm, suggesting a role for SsSvf1 in lipid metabolism in the cytoplasm. It is interesting to note that lipocalin has been reported to be a protective factor against H_2_O_2 _toxicity (Roudkenar *et al*., 2008).

Svf1 inhibited ROS generation in *S. cerevisiae*, and the cells lacking *SVF1* have increased levels of ROS (Brace *et al*., 2005). Our results showed that silencing of *SsSvf1* led to overproduction of ROS in the hyphae suggesting that *SsSvf1* also regulates ROS generation of *S. sclerotiorum*. To date, only a few genes have been demonstrated to play important roles in regulating the internal redox environment in *S. sclerotiorum*. NADPH oxidases (Nox) are a primary source for endogenous generators of ROS (Nauseef, 2008; Takemoto *et al*., 2007). Silencing of the *S. sclerotiorum* Nox‐encoded gene *Ssnox1* expression can impair superoxide production (Kim *et al*., 2011). The γ‐glutamyl transpeptidase (Ggt) catalyzes the first step in glutathione (GSH) metabolism and recycling; it is an important factor in maintaining cellular redox homeostasis (Lee and Bostock, 2007). Deletion of the Ggt‐encoded gene *Ss‐Ggt1* in *S. sclerotiorum* resulted in H_2_O_2_ being hyper‐accumulated in sclerotia (Li *et al*., 2012). However, more evidence is required to elucidate the mechanism whereby *SsSvf1* is involved in regulating ROS generation in *S. sclerotiorum*, because SsSvf1 exhibits no similarity with any known enzymes.

*S. sclerotiorum* secretes OA to elicit a host programmed cell death (PCD) response, which provides nutrients that are beneficial for this necrotrophic fungal pathogen (Kim *et al*., 2008). The PCD response requires ROS generation, the production of which may be stimulated by necrotrophs (Marino *et al*., 2012). Enhanced ROS generation stimulates the necrosis induced by *S. sclerotiorum* (Govrin and Levine, 2000). Thus, ROS modulation is critical for successful infection by this fungus. In this research, the virulence of *SsSvf1* gene‐silenced strains were impaired on different hosts, while showing partial recovery on hosts in which the ROS generation was inhibited. Although DPI was also used to inhibit NADPH oxidases in *B. cinerea* and *S. sclerotiorum*, direct evidence that it can inhibit ROS production in these two fungi cannot be found (Kim *et al*., 2011; Segmüller *et al*., 2008). Our results suggest that SsSvf1 plays an important role in anti‐oxidation during *S. sclerotiorum* infection. Recent evidence indicates that *S. sclerotiorum* has a short biotrophic phase during the early stages of infection, and it uses OA to suppress the oxidative burst during initial stages (Kabbage *et al*., 2015; Williams *et al*., 2011). The inhibition of ROS generation by DPI can benefit the fungus during compatible interactions, although more evidence is needed.

As multi‐cellular infectious structures, compound appressoria play important roles in *S. sclerotiorum* pathogenesis (Huang *et al*., 2008; Liang *et al*., 2015a, b; Xiao *et al*., 2014). Compound appressoria might help the fungus adhere to and penetrate the host cuticle and also secrete enzymes and toxins (Huang *et al*., 2008; Jamaux *et al*., 1995). In *S. sclerotiorum*, appressorium development might depend on the cAMP‐PKA signalling pathway and be associated with OA accumulation (Jurick and Rollins 2007; Liang *et al*., 2015a, b). In this research, *SsSvf1* gene‐silenced strains showed impaired activity in appressorial formation, and their virulence was partially restored on wounded leaves of rapeseed, indicated that *SsSvf1* was important for the appressoria formation. Svf1 function was only reported in *S. cerevisiae*, a non‐pathogenic and non‐appressoria forming fungus that did not form appressorium. Thus, the mechanism of *SsSvf1* involved in appressorial formation is still unknown. A closer investigation showed that *SsSvf1* gene‐silenced strains were more sensitive to some cell wall damaging agents suggesting a possible defect in the cell wall components of the hyphae for these strains. The combined evidence suggests that the reduced efficiency in appressorial formation is due to the impaired integrity of the hyphae cell wall. Defects in the cell wall components influencing appressorial formation have been reported for many important pathogenic fungi, including *Magnaporthe grisea* (Jeon *et al*., 2008; Skamnioti *et al*., 2007; Xu, 2000) and *Colletotrichum graminicola* (Albarouki and Deising 2013).

The molecular mechanism of *SsSvf1* in maintaining the cell wall integrity of hyphae in *S. sclerotiorum* remains unclear. *S. cerevisiae Svf1* affected the localized generation of a pool of phytosphingosine, which might activate the Ypk1p pathway that play a role in the cell wall integrity pathway (Brace *et al*., 2007; Liu *et al*., 2005; Schmelzle *et al*., 2002). Our results showed that the *SsSvf1* gene‐silenced strains were highly sensitive to CR but exhibited a similar level of sensitivity to CFW versus the wild‐type strain. The CFW inhibits chitin polymer assembly in the cell wall, but CR affects glucan polysaccharide assembly (Daher *et al*., 2011; Puttikamonkul *et al*., 2010; Ram and Klis, 2006). The results suggest that the chitin content was likely unaffected in *SsSvf1* gene‐silenced strains. Glucan is an important component of the cell wall and is critical for maintaining cell integrity (Ram *et al*., 1998). However, additional studies on the alteration of glucan in *SsSvf1* gene‐silenced strains are needed.

In summary, our results demonstrate that *SsSvf1* encodes a *S. cerevisiae* survival factor 1 homologue in *S. sclerotiorum*. The gene was characterized and found to play a crucial role in response to oxidative stress, cell wall integrity and pathogenicity of *S. sclerotiorum*. This finding helps to explain the pathogenicity of *S. sclerotiorum* and molecular mechanism involved in the antioxidant response in plant fungal pathogens.

## Experimental Procedures

### Fungal strains and cultured conditions

*S. sclerotiorum* strain 1980 was used as the wild‐type strain and was routinely cultured on PDA medium. Transformant strains were cultured on PDA with 100 μg/mL hygromycin B (Calbiochem, San Diego, CA).

### RNA isolation and cDNA synthesis

Total RNA was extracted from frozen mycelium of the wild‐type strain or transformants, or inoculated *A. thaliana* leaf sample with Rrizol reagent (Huashun Biogengineering Co, Shanghai, China) according to the manufacturer's instructions. The total RNA was then treated with DNase 1 (RNase free) (Takara, Dalian, China) to remove DNA contaminants. Approximately 1 μg of treated RNA was used to synthesize the cDNA with the ReventAid^TM^ First Strand cDNA Synthesis Kit (MBI Fermentas, Flamborough, ON, Canada) following the manufacturer's instructions.

### Real‐time RT‐PCR

To determine the expression levels of *SsSvf1*, real‐time RT‐PCR using SYBR Green I technology on a CFX96^TM ^Realtime System (BioRad, Hercules, CA, USA) was performed. The housekeeping gene *Tub1* encoding β‐tubulin was used as an internal control and amplified with Rt‐tubfp/Rt‐tubrp primers. The primer pair Rt‐Svf1fp/Rt‐Svf1rp was designed according to the cDNA sequence of *SsSvf1*. Real‐time RT‐PCR was conducted in a 20 μL reaction mixture containing 10 μL of SYBR Green Realtime PCR Master Mix (Toyobo, Osaka, Japan), 4 pmoL concentration of each primer, 1 μL of cDNA, and nuclease‐free water. The following amplification programme was applied: 95 °C for 2 min (1 cycle), followed by 95 °C for 20 s, 57 °C for 15 s and 72 °C for 20 s (40 cycles). Each sample was analysed in three biological replications, and the average cycle threshold was calculated to evaluate the relative expression. The primers used here are shown in Table 1.

**Table 1:** Sequence of primers used in the research.

Primer	Primers sequence (5′‐3′)
Rt‐tubfp	GTGAGGCTGAGGGCTGTGA
Rt‐tubrp	CCTTTGGCGATGGGACG
Rt‐Svf1fp	TGTCGTAGGAACTGAGGAGCC
Rt‐Svf1rp	GTACTCTCTTGAGCCTTCCATTG
SiSvf1Xho I	CCGCTCGAGTCTGATGCGCTCGAAAACTATG
SiSvf1Hind III	CGCAAGCTTATGGCGTGAATAAAAATACCGG
SiSvf1Kpn I	CGGGGTACCTCTGATGCGCTCGAAAACTATG
SiSvf1Bgl II	GGAAGATCTATGGCGTGAATAAAAATACCGG

### *SsSvf1* gene‐silenced vector construction and transformant

The *SsSvf1* gene‐silencing vector was constructed based on plasmid pSilent‐1 (Nakayashiki *et al*., 2005). To amplify the sense and antisense fragments of *SsSvf1*, the primers SiSvf1Xho I/SiSvf1Hind III (sense fragment) and SiSvf1Kpn I/SiSvf1Bgl II (antisense fragment) were used. The amplified sense and antisense fragments were inserted into the corresponding multiple cloning sites of pSilent‐1 to generate the RNA silencing vector pSiSvf1. The vector was then linearized with *Spe* I and used to transform the wild‐type strain of *S. sclerotiorum* according to the method of Rollins (2003).

### ROS detection assay

The ROS detection assay was performed according to Kim *et al*. (2011). The strains were inoculated on PDA plates for 2 days and the plates were then immersed in 0.5 mg/mL NBT (10 mM potassium phosphate buffer, pH 7.5) aqueous solution for 2 h with gentle shaking. The hyphae were observed with light microscopy. ROS accumulation in hyphae tips was quantified via mean pixel intensity with ImageJ software according to Egan *et al*. (2007).

### Pathogenicity assay

*A. thaliana* Columbia‐0 and *Brassica napus* Zhongyou 821 were used for the pathogenicity assay according to Yu *et al*. (2017). To analyse the pathogenicity of the strains on host plants in which ROS production was inhibited, leaves of *B. napus* were sprayed with 5 μM DPI dissolved in water and then were inoculated with mycelia‐colonized agar (6 mM in diameter) obtained from the growing colony margins of the strain. The lesions were measured at 48 hpi. In each experiment, each strain was inoculated on five plants or leaves and the experiment was repeated three times.

### OA concentration assays

The OA concentration assay was performed according to Yu *et al.* (2017) with minor modifications. The wild‐type strain and SiSvf1‐213 were cultured on PDA medium for 2 days. Subsequently, four mycelium plugs (6 mM in diameter) of each strain were cultured in 50 mL of potato dextrose broth (PDB) for 3 days with shaking at 150 rpm. OA accumulation was determined by high‐performance liquid chromatography according to Zhang *et al.* (2010). The concentration was expressed as milligrams of OA per gram of dry mycelia. Each strain was repeated three times.

### Compound appressorium assay

To assay the compound appressorium formation of the wild‐type and *SsSvf1* gene‐silenced strains, 2‐day‐old culture plugs (6 mM in diameter) were inoculated onto parafilm and rapeseed leaves. The plugs were removed at 8 hpi for those on parafilm. To observe the appressorium, the parafilm surface were stained with 5% trypan. The leaves were cleared with ethanol/acetic acid (3:1 v/v) solution for 12 h and then stained with 5% trypan blue for 12 h.

### Cell wall integrity assay

The sensitivity of the hyphal growth to different cell wall inhibitors were tested to evaluate the cell wall integrity of the wild‐type and *SsSvf1* gene‐silenced strains. Two‐day‐old culture plugs (6 mM in diameter) of each strain were inoculated onto PDA amended with 0.02% SDS, 400 μg/mL CR, or 200 μM CFW. The colony diameters were measured after incubation for 36 h to determine the inhibition of hyphal growth; unmodified plates served as the control.

## Supporting information


**Fig. S1** Expression level of *SsSvf1* in different isolates containing pSiSvf1. *Tub1* gene in each strain was the internal control. The relative expression of *SsSvf1* in the wild‐type strain was set as one. Bars indicate standard deviation.

**Fig. S2** Oxalic acid (OA) accumulation in wild‐type strain and SiSvf1‐213. Each strain was cultured in potato dextrose broth (PDB) for 3 days, and the resulting liquid culture was analysed for OA accumulation.
