# Supplementary figures and images for: Survival factor 1 contributes to the oxidative stress response and is required for full virulence of Sclerotinia sclerotiorum
Source: Mol Plant Pathol. 2019 May 9;20(7):895–906. doi: 10.1111/mpp.12801 (PMC6589728; doi:10.1111/mpp.12801)

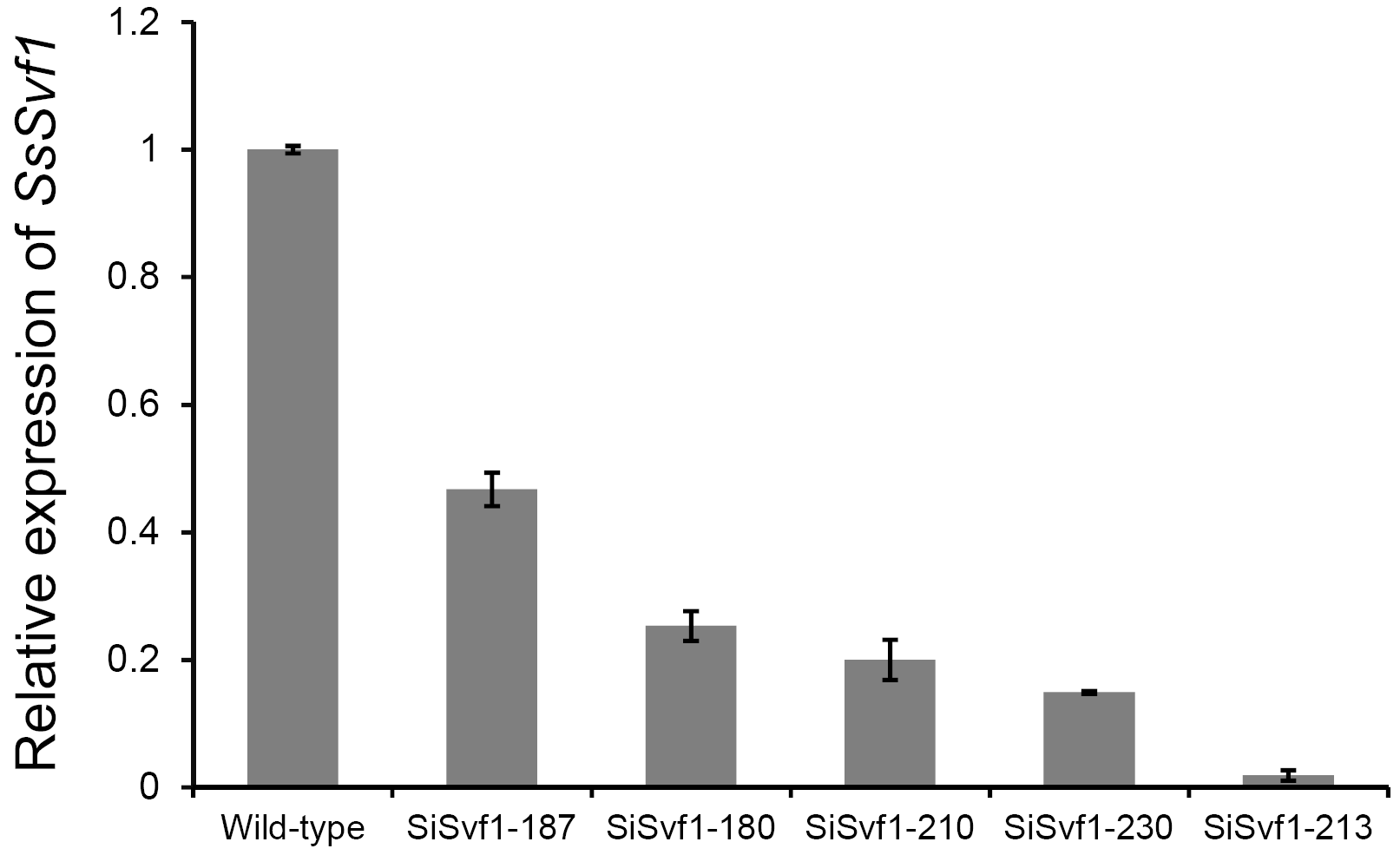

Supplement: Supplementary file 1 — Fig. S1 Expression level of SsSvf1 in different isolates containing pSiSvf1. Tub1 gene in each strain was the internal control. The relative expression of SsSvf1 in the wild‐type strain was set as one. Bars indicate standard deviation. [file MPP-20-895-s001.tif]

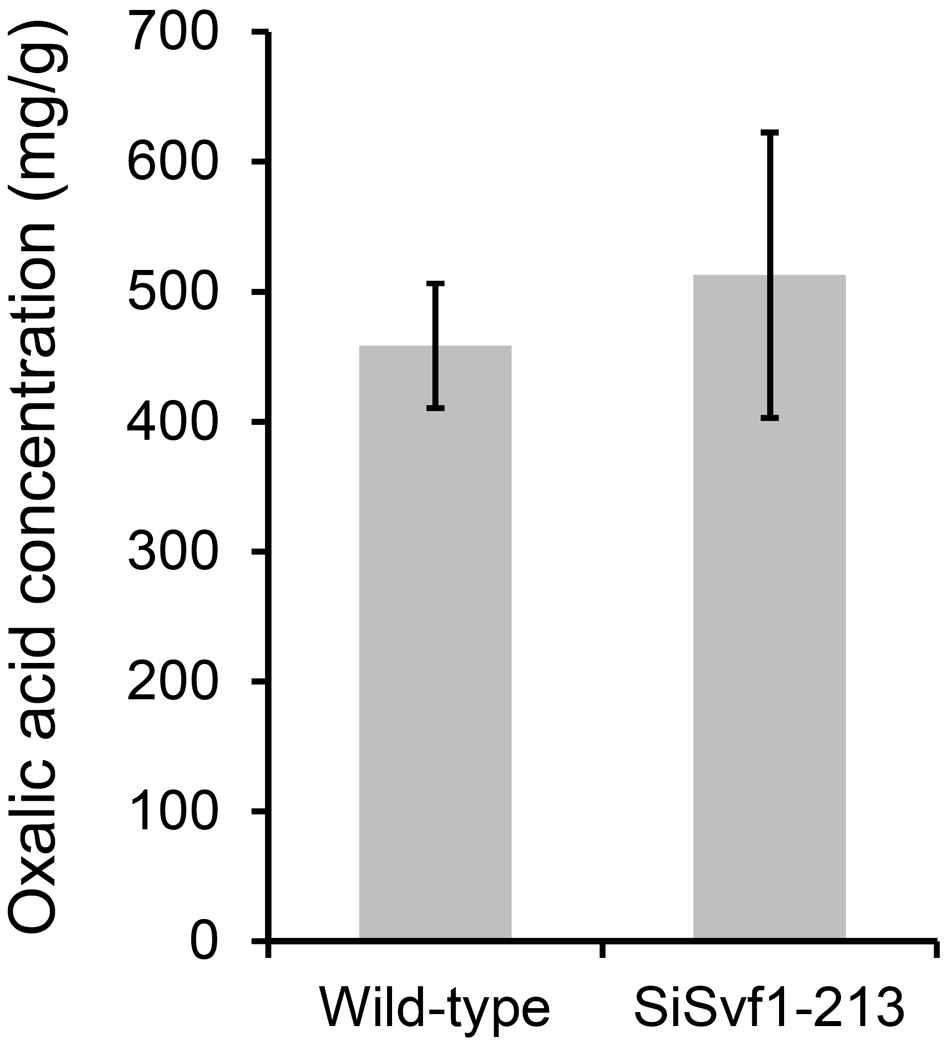

Supplement: Supplementary file 2 — Fig. S2 Oxalic acid (OA) accumulation in wild‐type strain and SiSvf1‐213. Each strain was cultured in potato dextrose broth (PDB) for 3 days, and the resulting liquid culture was analysed for OA accumulation. [file MPP-20-895-s002.tif]
